# Trajectory correction enables free-running chemical shift encoded imaging for accurate cardiac proton-density fat fraction quantification at 3T

**DOI:** 10.1016/j.jocmr.2024.101048

**Published:** 2024-06-13

**Authors:** Pierre Daudé, Thomas Troalen, Adèle L.C. Mackowiak, Emilien Royer, Davide Piccini, Jérôme Yerly, Josef Pfeuffer, Frank Kober, Sylviane Confort Gouny, Monique Bernard, Matthias Stuber, Jessica A.M. Bastiaansen, Stanislas Rapacchi

**Affiliations:** a Aix-Marseille Univ, CNRS, CRMBM, Marseille, France; b APHM, Hôpital Universitaire Timone, CEMEREM, Marseille, France; c Siemens Healthcare SAS, Saint-Denis, France; d Department of Diagnostic and Interventional Radiology, Lausanne University Hospital, Lausanne, Switzerland; e Department of Diagnostic, Interventional and Pediatric Radiology (DIPR), Inselspital, Bern University Hospital, University of Bern, Bern, Switzerland; f Translation Imaging Center (TIC), Swiss Institute for Translational and Entrepreneurial Medicine, Bern, Switzerland; g Advanced Clinical Imaging Technology, Siemens Healthineers International AG, Lausanne, Switzerland; h Center for Biomedical Imaging, Lausanne, Switzerland; i Siemens Healthcare, MR Application Development, Erlangen, Germany

**Keywords:** Gradient impulse response function, Cardiac fat-water MRI, Free-running MRI, Epicardial adipose tissue

## Abstract

**Background:**

Metabolic diseases can negatively alter epicardial fat accumulation and composition, which can be probed using quantitative cardiac chemical shift encoded (CSE) cardiovascular magnetic resonance (CMR) by mapping proton-density fat fraction (PDFF). To obtain motion-resolved high-resolution PDFF maps, we proposed a free-running cardiac CSE-CMR framework at 3T. To employ faster bipolar readout gradients, a correction for gradient imperfections was added using the gradient impulse response function (GIRF) and evaluated on intermediate images and PDFF quantification.

**Methods:**

Ten minutes free-running cardiac 3D radial CSE-CMR acquisitions were compared in vitro and in vivo at 3T. Monopolar and bipolar readout gradient schemes provided 8 echoes (TE1/ΔTE = 1.16/1.96 ms) and 13 echoes (TE1/ΔTE = 1.12/1.07 ms), respectively. Bipolar-gradient free-running cardiac fat and water images and PDFF maps were reconstructed with or without GIRF correction. PDFF values were evaluated in silico, in vitro on a fat/water phantom, and in vivo in 10 healthy volunteers and 3 diabetic patients.

**Results:**

In monopolar mode, fat-water swaps were demonstrated in silico and confirmed in vitro. Using bipolar readout gradients, PDFF quantification was reliable and accurate with GIRF correction with a mean bias of 0.03% in silico and 0.36% in vitro while it suffered from artifacts without correction, leading to a PDFF bias of 4.9% in vitro and swaps in vivo. Using bipolar readout gradients, in vivo PDFF of epicardial adipose tissue was significantly lower compared to subcutaneous fat (80.4 ± 7.1% vs 92.5 ± 4.3%, *P* < 0.0001).

**Conclusions:**

Aiming for an accurate PDFF quantification, high-resolution free-running cardiac CSE-MRI imaging proved to benefit from bipolar echoes with k-space trajectory correction at 3T. This free-breathing acquisition framework enables to investigate epicardial adipose tissue PDFF in metabolic diseases.

## Graphical abstract

**Figure:**
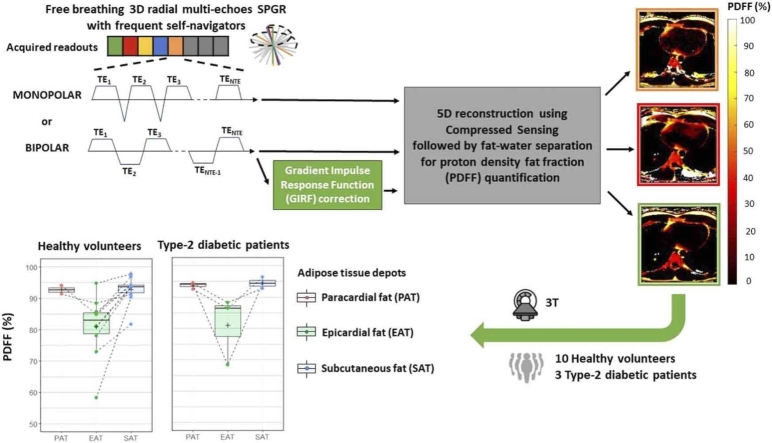

## 1 Background

There is a growing interest in probing epicardial adipose tissue (EAT), a metabolic fat surrounding the heart, that has been shown to influence pathophysiological pathways toward cardiovascular degradation in metabolic diseases [1]. Interestingly, EAT can participate in cardiac risks both from its volume, accumulating progressively all around the heart and from its composition, changing from a “brown-like” fat, deemed “beige,” to a more deleterious “white” [1]. High-resolution mapping of fatty depots is preferentially performed with chemical shift encoded (CSE) cardiovascular magnetic resonance (CMR) [2], which provides quantification of both proton-density fat fraction (PDFF) and T_2_* decay. Those biomarkers have been shown to distinguish the color feature of adipose tissue (brown or white) [3], [4], which informs about the metabolic activity of the fat. Currently, in CMR, PDFF measurements have helped identifying myocardial fatty infiltration and lipomas [5]. Meanwhile, T_2_* mapping received increasing interest in community and consensus statements [6], [7] for the assessment of hemorrhage [8] and iron deposition [9].

However, three-dimensional (3D) CSE-CMR is challenging in the heart due to cardiac and respiratory motion. Most 3D approaches [10], [11] have been triggered to a single cardiac phase (end-diastole) to minimize cardiac motion artifacts, while navigator gating is used to reduce respiratory motion artifacts. Recently at 1.5T, cine 3D Dixon in conjunction with navigator gating [12] has been developed. Resolving cardiac motion allowed to evaluate EAT in systole, which minimizes epicardial border partial volume effects. While benefiting from increased field-strength boosting signal-to-noise ratio (SNR) and fat sensitivity, CSE-CMR at 3T remains challenging due to rapid phase accrual between water and fat and the concomitant inhomogeneous B_0_ field [13] in the heart. Currently, single phase or cine [14], [15] CSE-CMRI [16] with navigator gating has been proposed at 3T, with the limitation to fat imaging without PDFF quantification due to the use of fewer echoes.

The free-running framework is a recent approach for high-resolution functional cardiac imaging acquiring 3D radial samples using a phyllotaxis trajectory with complete respiratory and cardiac self-gating and combined with a multidimensional compressed sensing reconstruction [17]. It circumvents the need for respiratory navigation by using a self-gated superior-inferior projection, which can also be employed for cardiac gating. This framework has demonstrated its capacity for motion-resolved high-resolution CMR, particularly to target demanding coronary imaging [18], five-dimensional (5D)-Flow MRI [19], and more recently fat-fraction mapping at 1.5T [20]. Interestingly, epicardial fat imaging requires high-resolution imaging, and its visualization is commonly preferred in systole [21], due to the myocardium pulling the thin fat layer away from the pericardium sack, thus enhancing EAT delineation and reducing partial volume effects. We hypothesized that the free-running framework lays the ground for high-resolution quantitative cardiac CSE-CMR at 3T.

We hypothesize that, at 3T, bipolar readout gradients allow for shorter echo spacing, which inherently provide more accurate PDFF quantification than monopolar readout gradients with rewinding gradients. However, bipolar CSE-CMR imaging suffers from distortions between odd and even echoes, in particular using non-Cartesian CSE-CMR imaging [22]. These distortions arise due to gradient imperfections that are more important for the rapidly switching gradients required for bipolar echoes. These distortions were also more severe since they vary for each k-space line in the free-running non-Cartesian sampling, thus impacting image reconstruction at the gridding stage. Recently, correction strategies using the gradient impulse response function (GIRF) [23], [24] have emerged as a generic and efficient solution. First, the system's three-axis GIRF is fully characterized. Second, the actual gradient shapes played during any sequence are evaluated with GIRF and integrated over time. Eventually, the sampled k-space trajectory can be corrected for image reconstruction [25].

Thus, the free-running cardiac CSE-CMR sequence was implemented at 3T with both monopolar and bipolar readout gradients. For bipolar readout gradients, image reconstruction was extended and evaluated with and without k-space trajectory correction using GIRF. Finally, high-resolution quantitative cardiac CSE-CMR at 3T was demonstrated in healthy volunteers and diabetic patients.

## 2 Methods

### 2.1 Numerical simulation

Synthetic CSE-CMR data *y* were simulated with parameters matching practical acquisitions, for monopolar readouts: echo time number/first value/spacing: NTE/TE1/ΔTE = 8/1.16 ms/1.96 ms, and for bipolar readouts: NTE/TE1/ΔTE = 13/1.12/1.07 ms, and a noisy fat-water signal model defined as:

$$y=\left(W+F\sum_{m=1}^{M}\alpha_{m}e^{i\omega_{m}t}\right)e^{i\varphi_{0}+\left(i{2\pi f}_{0}-{R_{2}}^{*}\right)t}+\eta \left(t\right)$$

with W and F corresponding to normalized water and fat magnitude signals, $f_{0}$ the off-resonance frequency, $\varphi_{0}=\;30{}^{\circ}$ the common initial phase which holds only for low flip angles [26], R_2_* = 50 s^−1^ the transversal decay, η(t) the complex Gaussian noise (with SNR = 10 or 50) and $\alpha_{m}$, $e^{i\omega_{m}}$ the relative normalized amplitudes ($\sum_{m}^{M}\alpha_{m}=1$), and frequency offsets of a 10-peaks resolved subcutaneous fat spectrum [27], [28], respectively. The virtual CSE-CMR data were synthesized as volumes using an open-source toolbox [28] with along the x-axis, PDFF varied from 0% to 100% with 1% step, along the y-axis, f_0_ was uniformly distributed from −200 Hz to 200 Hz with 4 Hz step, and the z-axis consisted in 100 repetitions. Synthetic volumes were normalized based on 99% of the maximum of the first echo. PDFF and R_2_* quantification precision were evaluated on the noisy simulated data.

### 2.2 GIRF measurement

The system-specific GIRF was measured using the two off-centered slices method on a spherical phantom placed at isocenter [29], [30]. The free-running sequence gradient waveforms (Fig. 2A) were corrected with the GIRF measurements using multiplication in the frequency domain. Temporal integration provided the actual k-space trajectory (Fig. 2B).

### 2.3 CMR acquisition

All data were acquired on a 3T MRI system (MAGNETOM Vida, equipped with 60 mT/m and 200 mT/m/s gradients, exact field strength 2.89T, Siemens Healthcare, Erlangen, Germany) with the spine coil array and an 18-channel body coil array (providing between 24 and 30 channels for cardiac imaging) unless specified otherwise. A research 3D radial spoiled gradient echo sequence was implemented with multiple echoes and a phyllotaxis trajectory for integration with the free-running framework [17].

The multiple echoes were designed to fit within a single repitition time (TR) of 15 ms, which has been shown to be sufficient for quantitative characterization of abdominal fat [31], leading to 13 echoes (TE1/ΔTE = 1.12/1.07 ms) in bipolar mode and 8 echoes (TE1/ΔTE = 1.16/1.96 ms) in monopolar mode. Each acquisition lasted 10 min 38 s acquisition, with the following parameters: 40,014 radial views per echo acquired in a phyllotaxis segmented acquisition with 13 TR per segments, and 3078 segments total with FOV = (220 mm)^3^ at isotropic (1.5 mm)^3^ resolution, flip angle 5°, receiver bandwidth 1510 Hz/px for all echoes.

### 2.4 In vitro: fat water phantom

Validation of the PDFF quantification was performed on a fat-water phantom. Eight 50 mL vials were custom-built using documented recipes [32] to obtain increasing fat fractions of water-peanut oil emulsions, which were imaged using a 20-channel head coil. The exact fat/water volume percentages were determined with magnetic resonance spectroscopy, providing reference values of 0%, 8.5%, 20%, 37%, 63.2%, 85.4%, 92.1%, and 100%. Spectroscopy data were acquired separately for each individual phantom vial in the 3T MRI system using a non-localized free-induction decay (FID) sequence with TR = 8000 ms. Phantom vials were bundled and aligned along the static magnetic field direction to maximize B0 homogeneity, and iterative shimming was performed before acquisition. Synthetic respiratory and cardiac signals were generated to obtain 4 respiratory bins and 10 cardiac phases. SNR was measured in the vials over a 3D region of interest and defined as the mean signal divided by the standard deviation.

### 2.5 Study population

Ten healthy volunteers (age: 36 ± 12 years; body mass index (BMI): 22.9 ± 1.7 kg/m^2^; male\female: 8\2) and 3 diabetic patients (age: 65 ± 5 years; BMI: 29.4 ± 9.9 kg/m^2^; male\female: 0\3) were recruited in this study after informed consent.

### 2.6 Image reconstruction and quantitative fat-water mapping

Cardiac and respiratory motion signals were extracted and interpolated from the first echo of the superior-inferior projections [17] that occurred at the first TR of each segment. Data were binned in 4 respiratory phases and 100-ms-wide cardiac phases, resulting in subject-specific number of cardiac phases varying from 7 to 12 depending on their heart rate. The 6D binned k-space data were reconstructed using the free-running compressed sensing framework [17], [33]. To alleviate memory handling, multi-echo 5D images (x-y-z, cardiac and respiratory dimensions) were reconstructed on a per-echo basis, after singular value decomposition (SVD) coil compression using a compressed sensing [34], [35] algorithm that exploited sparsity along spatial, cardiac, and respiration dimensions. The SVD coil compression employed a 2% threshold on the eigenvalues, reducing the number of compressed coils to approximately 12. The optimization problem was solved using Alternating Direction Method of Multipliers (ADMM) algorithm [36] with total variation regularization weights along spatial (λ_s_), cardiac(λ_c_), and respiratory (λ_r_) dimensions, the penalty term of the augmented Lagrangian (ρ) and the number of ADMM iterations (N_iter_) set to λ_s_ = 0.0015, λ_c_ = 0.0075, and λ_r_ = 0.005, ρ = 0.06, and N_iter_= 10. Based on the 3D radial Nyquist criterion, individual free-running CSE-CMR data were accelerated by a factor R = 26 for 8 cardiac phases and 4 respiratory states.

Complex images from each bin were processed for fat-water separation using Iterative Decomposition of water and fat with Echo Asymmetry and Least square Estimation (IDEAL) method with constrained extension [37]. Computations were performed in MATLAB (R2019b, Mathworks Inc., Natick, Massachusetts) with a computer equipped with 120 GB RAM, a graphics processing unit (GPU) (Quadro RTX 6000, 24 GB, Nvidia, San Franscisco, California), and 48 CPUs (Xeon Gold 5220R, 2.20 GHz, Intel, San Fransisco, California).

### 2.7 Evaluation metrics and statistical analysis

The difference between monopolar and bipolar acquisitions was evaluated in silico and in vitro using standard metrics (bias, precision) on the respective PDFF maps. PDFF in different regions of interest (ROI) was measured to evaluate the performances of the proposed correction in vivo: right ventricle (RV) and left ventricle (LV), septum, liver, bone marrow, subcutaneous adipose tissue (SAT), EAT, and paracardial adipose tissue (PAT). The expected PDFFs of the different regions are the following: with negligible PDFFs (∼0%) are ventricles and septum, mid-low/mid-range PDFFs [0, 50]% are liver and bone marrow, and high PDFF (∼100%) is SAT whereas PDFF ranges of EAT, PAT are investigated in this study. ROI was manually segmented on CSE-CMR using im3Dtools v3.0 [38] in MATLAB.

Statistical analysis was conducted using R (version 3.6.3, R Foundation, Vienna, Austria) [39]. The metrics’ distribution normality was assessed using the Shapiro-Wilk test. Paired Wilcoxon signed rank and Wilcoxon rank sum tests were used to investigate significant differences for each quantitative parameter between monopolar and bipolar modes, the latest with and without correction.

## 3 Results

### 3.1 Numerical simulations

**Fig. 1:**
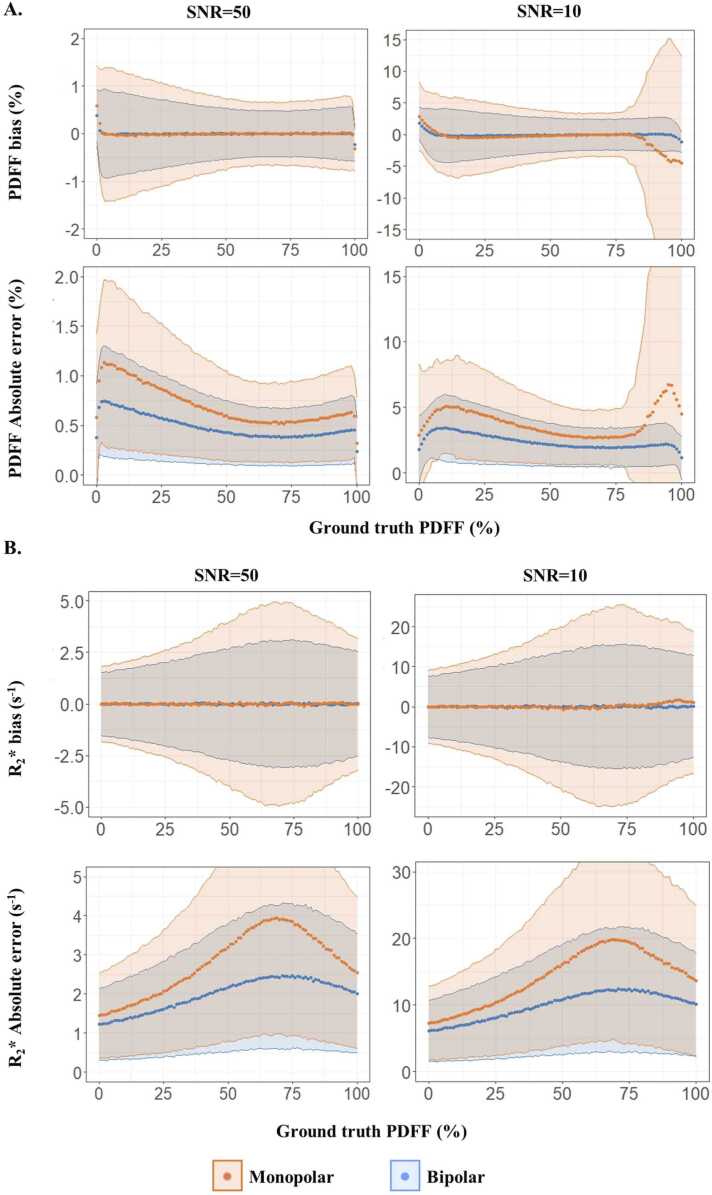
Comparison of PDFF (A) and R_2_* (B) bias and absolute error between 8 echoes in monopolar compared to 13 echoes in bipolar over synthetic CSE-CMR volumes with SNR = 50 and 10. Mean and standard deviation PDFF and R_2_* bias and absolute error were averaged along the $B_{0}$ off-resonance and repetition axes. *CSE* chemical shift encoded, *CMR* cardiovascular magnetic resonance , *PDFF* proton-density fat fraction, *SNR* signal-to-noise ratio.

PDFF and R_2_* quantification with an SNR = 50 was accurate (Fig. 1A) with both 13 and 8 echo times, with mean PDFF and R_2_* biases less than 0.01% and 0.01 s^−1^, respectively. However, with a lower SNR of 10, fat-water swaps were present for PDFF >85% in monopolar mode whereas PDFF quantification was still accurate for bipolar mode with a mean PDFF bias of 0.03%. Expectedly, PDFF absolute error significantly (*P* < 0.0001) decreased with 13 echoes compared to 8 echoes, from 0.7% to 0.5% and from 3.8% to 2.4% for SNR = 50 and 10, respectively. Additionally, R_2_* quantification proved to depend on PDFF with more imprecision in the 50% to 80% range. The maximum absolute errors were 4.0 and 19.8 s^−1^ with 8 echoes compared to 2.5 and 12.4 s^−1^ with 13 echoes at SNR = 50 and 10, respectively (Fig. 1B).

### 3.2 GIRF measurement results

**Fig. 2:**
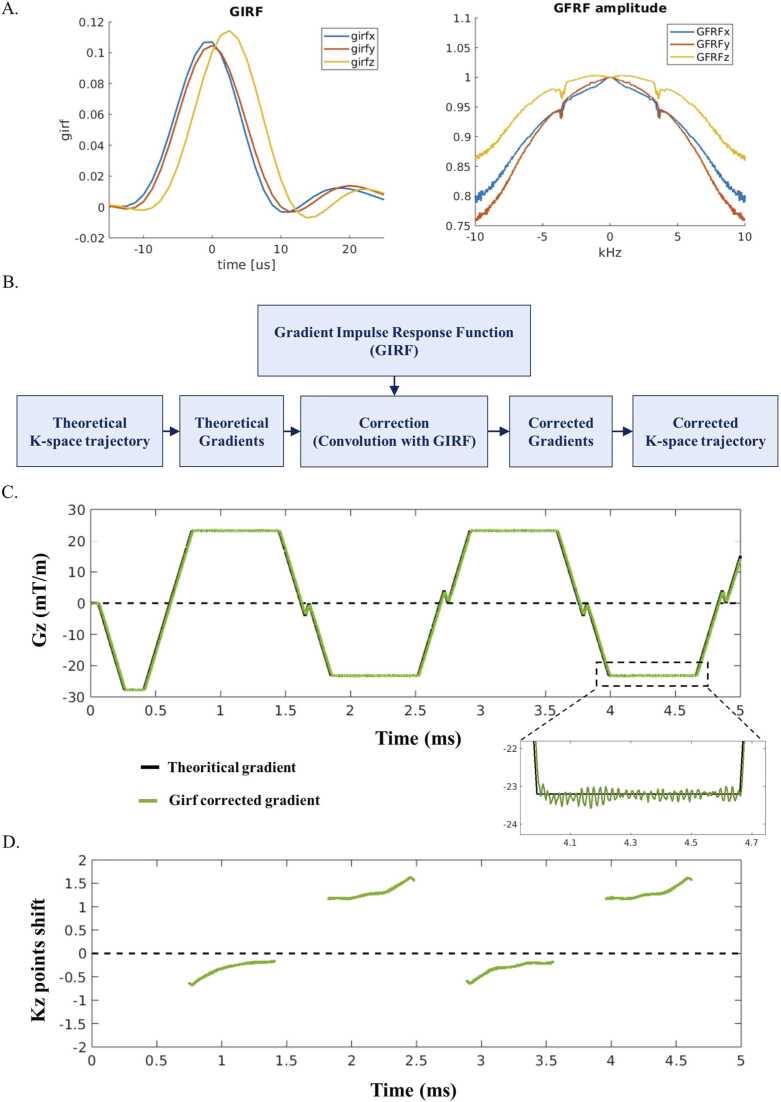
Trajectory correction using system-specific GIRF: (A) measurement of the GIRF and the magnitude of its Fourier transform (GFRF); (B) k-space trajectory correction pipeline (C) applied for the first four echoes in bipolar mode. (D) Converting distorted gradients into k-space trajectory showed up a mean shift superior to 1 for even echoes. GIRF-corrected trajectory prevents this error at the image reconstruction level. *GIRF* gradient impulse response function, *GFRF* gradient frequency response function.

Fig. 2 depicts the GIRF and the magnitude of its Fourier transform (gradient frequency response function or GFRF) in all three axes. The magnitude GFRF revealed the mechanical resonances of the gradient coils between 3.3 kHz and 4 kHz and a decreasing loss transfer function at high frequencies for all axes, impeding fast gradient switching such as that occurring in bipolar echoes mode. Moreover, the gradient response function is also dependent on the physical axis. The measured GIRF (Fig. 2A) exhibited a ∼2.5-µs delay in z axis whereas in frequency domain, GFRF along x and y gradient axes had a more degraded transfer function than along the z axis.

The GIRF correction revealed subtle oscillations on the gradient waveforms (Fig. 2C) and a mean shift of the sampled point in k-space trajectory by 1.28 and −0.34 points for even and odd echoes, respectively (Fig. 2D). Odd echoes were less impacted due to a built-in sequence calibration for the first echo and balanced errors between odd and even gradients.

### 3.3 Echo spacing scheme

**Fig. 3:**
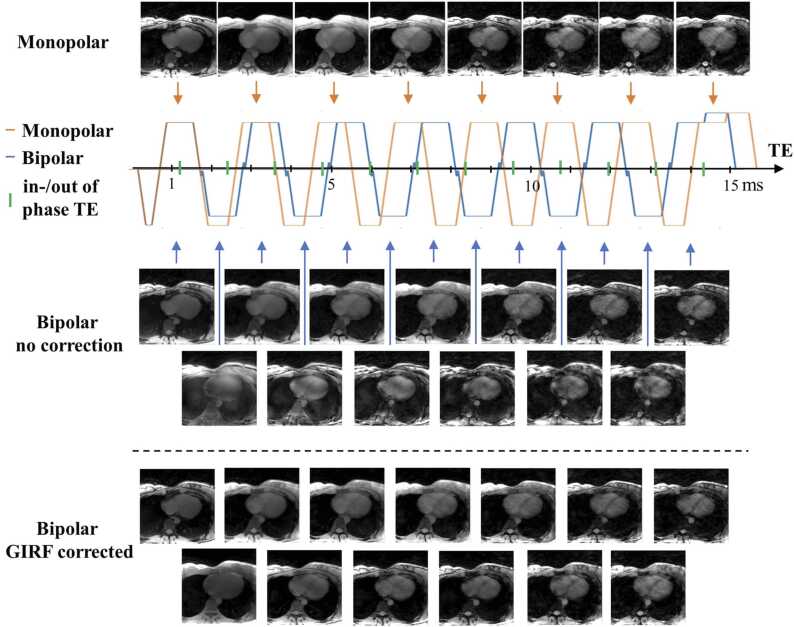
Free-running CSE-CMR echo times repartition depending on the multi-echo scheme, before and after GIRF correction. Monopolar echo spacing was longer than the fat-water in-phase/out-of-phase delay at 3T. In bipolar mode without correction, strong artifacts appeared on even echo images but after GIRF correction, image quality was restored. *CSE* chemical shift encoded, *GIRF* gradient impulse response function, *CMR* cardiovascular magnetic resonance.

At 3T, despite a high bandwidth and a modest matrix size, monopolar echoes (n = 8, minimum ΔTE = 1.96 ms) were unable to reach an echo spacing shorter than the required in-phase/out-of-phase delay (1.24 ms, green markers in Fig. 3). Only the bipolar echoes (n = 13) achieved a sufficiently short echo spacing (ΔTE = 1.07 ms).

However, in bipolar mode without correction, strong artifacts appeared between odd and even echoes (Fig. 3), including signal modulation over the image, signal losses in certain regions, and blurring of details. Artifacts disappeared after GIRF trajectory correction, providing consistent visualization throughout all echo times.

### 3.4 In vitro experiments

**Fig. 4:**
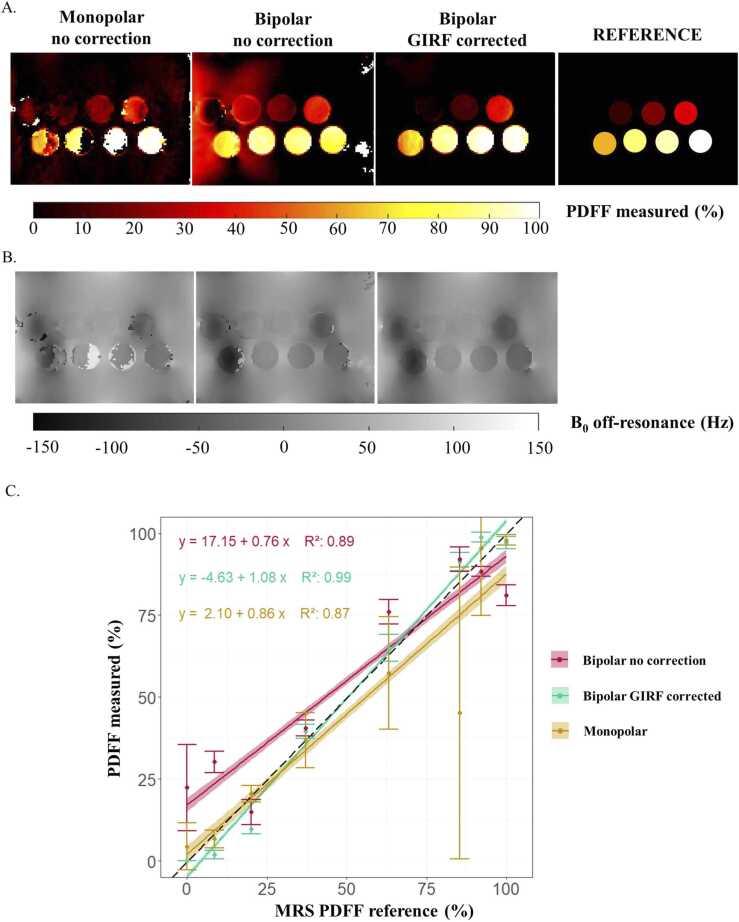
PDFF (A) and B_0_ off-resonance (B) measurements from in vitro experiments with monopolar and bipolar with or without GIRF correction. *GIRF* gradient impulse response function, *PDFF* proton-density fat fraction.

From the phantom experiments, SNR varied across vials between a minimum of 7 and a maximum of 43. Confirming simulation results, fat/water swaps were present in vials with PDFF >85% for the monopolar mode (Fig. 4). The corrected bipolar mode provided more accurate results with a mean bias of −0.4 ± 1.9% compared to monopolar mode with −5.3 ± 13.1%. Without correction, bipolar mode PDFF maps suffered from non-negligible bias (4.9 ± 4.4%). Linear regression results (Fig. 4C) showed that only corrected bipolar mode PDFF measurement was highly correlated to magnetic resonance spectroscopy (MRS) PDFF reference (corrected bipolar mode: R^2^ =0.99, non-corrected bipolar mode: R^2^ = 0.89, and monopolar mode: R^2^ = 0.87) with a slope close to 1 (corrected bipolar mode: 1.08, non-corrected bipolar mode: 0.76, and monopolar mode: 0.86).

### 3.5 In vivo experiments

**Fig. 5:**
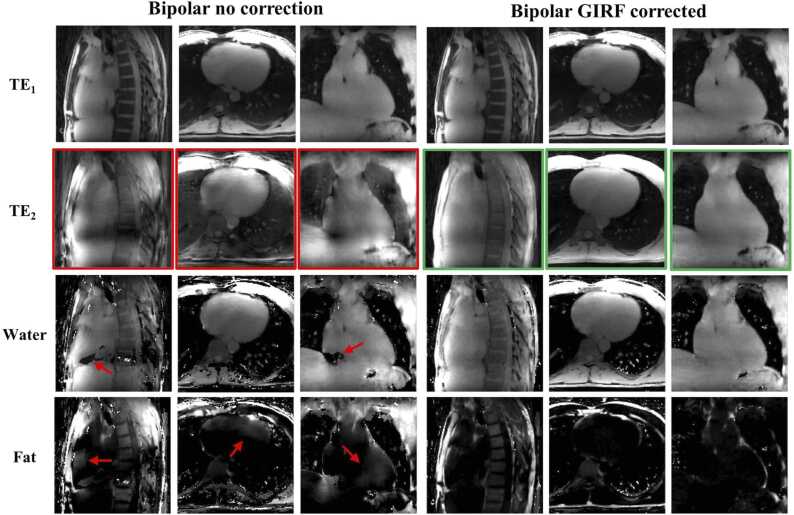
Impact of GIRF correction on free-running bipolar CSE-CMR on in-vivo data. Artifacted even echoes (second echo presented only) are highlighted in red resulting in severe swaps between fat and water reconstructed volumes, as pointed by the red arrows. Radial artifacts were reduced in GIRF-corrected images, corresponding to an almost in-phase image in vivo. *CSE* chemical shift encoded, *GIRF* gradient impulse response function, *CMR* cardiovascular magnetic resonance.

**Fig. 6:**
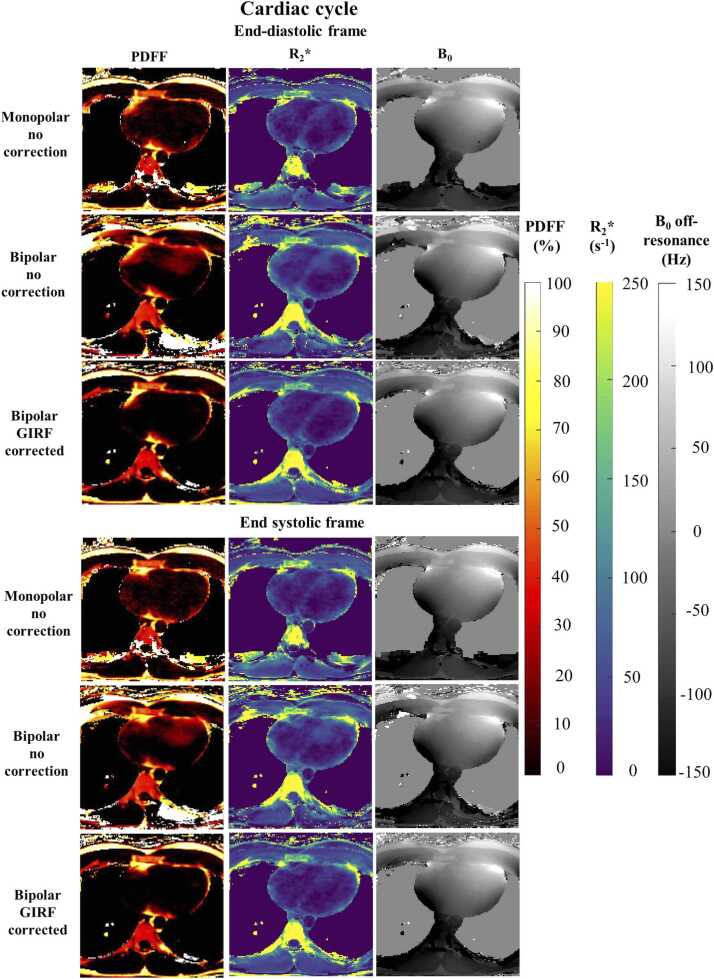
Quantitative PDFF and R_2_*, B_0_ off-resonance maps in axial view resulting from monopolar echoes or bipolar ones with/without GIRF correction revealed the propagation of image artifacts within the fat-water separation process. Across the different phases of the cardiac, inadequate monopolar echo spacing as well as uncorrected bipolar data led to spurious PDFF overestimation with fat-water swaps. *GIRF* gradient impulse response function, *PDFF* proton-density fat fraction.

Without GIRF correction, in vivo fat and water images suffered from blurring at the apex and around the atria and important swaps were observed between fat and water images (Fig. 5). These errors eventually corrupted corresponding quantitative maps (B_0_ offset, R_2_*, and PDFF) as observed along the cardiac and respiratory cycle (Figs. 6 7). For example, mean PDFF of 23.5% and 17.5% were measured in the LV and RV, respectively, regions with negligible expected adiposity (Fig. 8).

**Fig. 7:**
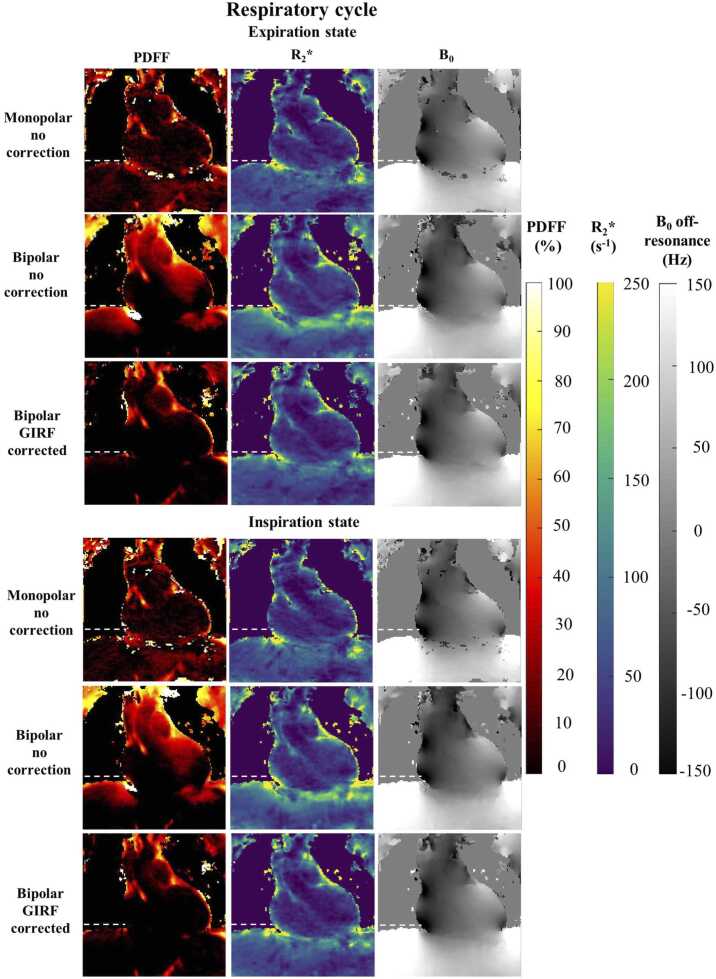
Quantitative PDFF and R_2_*, B_0_ off-resonance maps in coronal view resulting from bipolar with or without GIRF correction and monopolar echoes revealed the propagation of image artifacts within the fat-water separation process. Across the different phases of the respiratory cycle, inadequate monopolar echo spacing as well as uncorrected bipolar data led to spurious PDFF overestimation. The dotted white line represents the diaphragm level at expiration state. *GIRF* gradient impulse response function, *PDFF* proton-density fat fraction.

**Fig. 8:**
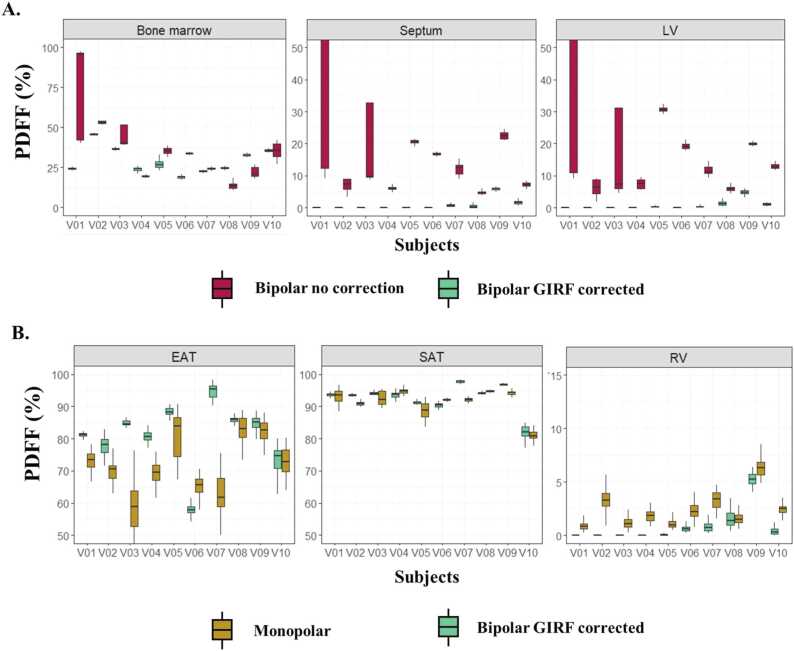
Comparison of PDFF measurements over different regions of interest across the volunteers between (A) bipolar echoes with or without GIRF correction, (B) monopolar or bipolar GIRF corrected echoes. Boxplots showed the consistency of PDFF measurements along cardiac and respiratory cycles. *GIRF* gradient impulse response function, *LV*: left ventricle, *PDFF* proton-density fat fraction.

On the contrary with GIRF correction, fat/water swaps were not observed, and water and fat regions were reliably distinguished in the heart throughout cardiac and respiratory states. Mean PDFF values in the ventricles were more realistic with a PDFF lower than 1% (LV and RV: 0.8%). In comparison, quantitative maps obtained with the monopolar acquisition still suffered from bias in PDFF and R_2_* quantification, with PDFF values superior to 2% (LV: 2.4%; RV: 2.5%).

In the healthy population, using GIRF-corrected bipolar readout gradients, epicardial fat had a significantly lower fat fraction than the subcutaneous fat (PDFF EAT = 80.4 ± 7.1% vs PDFF SAT = 92.5 ±4.3%, *P* < 0.0001). Although only present in two healthy volunteers, paracardial fat PDFF (92.5 ± 3.3%) tended to be superior to EAT PDFF. Bipolar GIRF-corrected PDFF values were more precise and homogenous across cardiac and respiratory cycles compared to the values from monopolar acquisitions, with a significantly (*P* < 0.0001) lower standard deviation (sd_PDFF_ = 1.23%, sd_PDFF_ = 2.70% monopolar, averaged across cardiac and respiratory cycles).

**Fig. 9:**
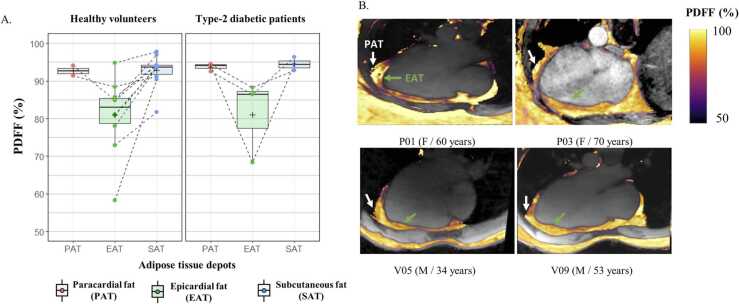
(A) PDFF in epicardial, paracardial, and subcutaneous fat across healthy volunteers and type-2 diabetic patients. (B) Comparison of PDFF maps between two patients (P01 and P03) and two healthy volunteers (V05 and V09) that presented both types of fat. PDFF maps are overlaid on anatomical images in four-chamber views for each corresponding subject. Arrows highlight each cardiac fat depot, green for EAT and white for PAT. *EAT* epicardial adipose tissue, *PAT* paracardial adipose tissue, *PDFF* proton-density fat fraction, *SAT* subcutaneous adipose tissue.

The preliminary results in three type-2 diabetic patients suggested that EAT fat fraction was also lower (81.0 ± 8.3%) compared to either subcutaneous fat (94.6 ± 3.9%) or paracardial fat (93.7 ± 3.8%) (Fig. 9).

## 4 Discussion

In this study, high-resolution high-precision 3D quantitative cardiac fat and water imaging was achieved using a free-running cardiac CSE-CMR framework at 3T. The full potential of free-running CSE-CMR at 3T could only be achieved with bipolar echoes, which required a correction with the system-specific GIRF characterization to prevent radial-trajectory image artifacts.

In monopolar mode, minimum inter-echo times (1.96 ms) were superior to in/out phase echo spacing (1.24 ms) for fat and water at 3T, which resulted in fat-water swaps in low SNR regions in silico. These swaps were confirmed in vitro in vials superior to 85% of fat fraction. As shown in Fig. 4B, B_0_ field map estimation errors could cause those swaps. However, with the bipolar readout gradients, 13 bipolar echoes could be acquired in a 15 ms single TR with ΔTE = 1.07 ms, and reliable PDFF measurement was reliable in simulation and in vitro and realistic in vivo with a PDFF lower than 1% in the region of negligible adiposity. Alternatively, an interleaved echo times approach (i.e., over two TR) could be envisioned, but this scheme is less efficient, would require two separate acquisitions unless the sequence was modified, and often requires an extra phase parameters to be adjusted to account for offsets between the two TR. Thus, it was not explored in this study.

EAT overload has been established as a biomarker of coronary heart disease (CHD). Its volumetric quantification using high-resolution 3D CSE-CMR has been already proposed in the past at 1.5T [12] and 7T [40]. Our approach can be distinguished from those earlier studies because it targets PDFF quantification of EAT with high precision. By acquiring 13 echoes, it allowed a differentiation of EAT PDFF, ranging around 80% and suggesting its “beige” color, from its neighbor paracardial fat PDFF, ranging close to 95% which corresponds rather to a typical white fat (Fig. 9). Quantitative PDFF might offer refine insights into the color features of EAT [3], [4]. The reduced PDFF of EAT could reflect different, individually or combined, specificities of this fat: its smaller fat droplets size, its higher perfusion from an embedded capillary network, and/or its metabolic activity involving a richer macromolecular content. Further investigation leveraging the 13 echoes could also provide valuable fatty acid composition characterization [41], [42].

Free-running cardiac CSE-MRI also provides with the full cardiac and respiratory cycles (Figs. 6 and 7, Supplementary Fig. 1), which offers multiple benefits: (1) imaging in systole is often preferred to study epicardial fat since the pericardium is thicker and better separates EAT from paracardial fat, (2) the respiratory phase with minimal B_0_ inhomogeneity can be chosen for analysis, limiting local signal loss and phase accumulation, and (3) variations of R_2_* along the cardiac cycle hold interest, in the myocardium to detect ischemia [43], but also between right and left ventricular blood pools to probe cardio-respiratory status [44]. Nevertheless, further evaluation along the cardiac cycle might be limited by the current self-gating sampling frequency (∼5 Hz) and would benefit from the use of the pilot tone sensor [20] for more accurate cardiac motion monitoring.

In addition to helping in the identification of myocardial fatty infiltration and cardiac lipomas [5], the isotropic 3D visualization also offers a substrate for volumetric evaluation of EAT overload, which could be automated as an extension of the recently proposed 2D EAT surface measurements using deep-learning image processing [45]. The automatic segmentation of EAT volume would also facilitate the quantitative PDFF assessment in CHD and cardio-metabolic diseases.

## 5 Limitations

Although different strategies could have been used for correcting k-space trajectory [46], [47], starting simply with constant and linear corrective factors, we opted for the GIRF method [25] which has proven to provide a more robust and precise correction. Beyond gradient imperfections, non-Cartesian k-space trajectories are also sensitive to gradient concomitant field effects and static field inhomogeneities. In our study, concomitant fields were considered negligible at 3T, although their correction [48] could be included along with the GIRF correction [49]. And due to the very short individual readout duration (∼1 ms), static field inhomogeneities have a minor impact on the trajectory, although a correction [50] embedded in the non-uniform fast Fourier transform could also be integrated in the free-running framework.

A major limitation of this work remains the prolonged reconstruction time. Indeed, due to the large amount of data (more than 20 GB) from each acquisition, each echo has been reconstructed independently with a computation time of 3 h 20 min per echo using the GPU. Fortunately, technical advances in image reconstruction hold hope for a significant reduction in this time.

Due to the high susceptibility-induced field of the lung, the B_0_ inhomogeneities field map had large variations influencing R_2_* quantification (Figs. 6 and 7). To compensate this confounding factor, it would be of interest to integrate a priori information of the scanner magnetic field distribution [51], [52] to improve the robustness of the B_0_ field map estimation and correct the R_2_* quantification [53].

## 6 Conclusions

This study aims at providing precise high-resolution PDFF quantification, achieved using free-running cardiac CSE-CMR at 3T. The acquisition of bipolar echoes proved to significantly outperform the monopolar echo mode but required k-space trajectory correction using the gradient system-specific GIRF characterization. It enabled us to investigate EAT PDFF, showing a significantly lower fat fraction (80.4 ± 7.1%) compared to subcutaneous fat (92.5 ± 4.3%) in a healthy subjects’ cohort.

## Funding

This project has received financial support from the CNRS through a Mission pour les initiatives transverses et interdisciplinaires (MITI) program and was performed within a laboratory member of France Life Imaging network (grant ANR-11-INBS-0006). J.B. received funding from the Swiss National Science Foundation (grant number PCEFP2_194296).

## Author contributions

**Josef Pfeuffer:** Writing – review and editing, Methodology. **Jérôme Yerly:** Writing – review and editing, Methodology. **Davide Piccini:** Writing – review and editing, Methodology. **Emilien Royer:** Writing – review and editing, Software, Resources. **Adèle L.C. Mackowiak:** Writing – review and editing, Methodology. **Stanislas Rapacchi:** Writing – review and editing, Writing – original draft, Validation, Supervision, Project administration, Methodology, Investigation, Formal analysis, Data curation, Conceptualization. **Thomas Troalen:** Writing – review and editing, Methodology. **Jessica A.M. Bastiaansen:** Writing – review and editing, Investigation, Conceptualization. **Pierre Daudé:** Writing – review and editing, Writing – original draft, Visualization, Validation, Software, Methodology, Investigation, Formal analysis, Data curation, Conceptualization. **Matthias Stuber:** Writing – review and editing, Project administration, Investigation, Conceptualization. **Monique Bernard:** Writing – review and editing, Resources, Project administration, Funding acquisition. **Sylviane Confort Gouny:** Writing – review and editing, Supervision, Project administration, Conceptualization. **Frank Kober:** Writing – review and editing, Conceptualization.

## Ethics approval and consent

The study was approved by the local ethic committees and all patients gave informed consent for their medical data to be used in this study.

## Consent for publication

The manuscript is approved by all authors for publication.

## Declaration of competing interests

The authors declare the following financial interests/personal relationships which may be considered as potential competing interests: Thomas Troalen, Josef Pfeuffer and Davide Piccini report a relationship with Siemens Healthcare that includes employment. Matthias Stuber reports a relationship with Siemens Healthcare that includes non-monetary research support. The other authors declare that they have no known competing financial interests or personal relationships that could have appeared to influence the work reported in this paper.

## Appendix A Supplementary material

Supplementary material. Fig. 1: Fat and water images with quantitative PDFF and R_2_*, B_0_ during the cardiac cycle in axial view resulting from bipolar with or without GIRF correction and monopolar echoes.
