# Supplementary figures and images for: Trajectory correction enables free-running chemical shift encoded imaging for accurate cardiac proton-density fat fraction quantification at 3T
Source: J Cardiovasc Magn Reson. 2024 Jun 13;26(2):101048. doi: 10.1016/j.jocmr.2024.101048 (PMC11269917; doi:10.1016/j.jocmr.2024.101048)

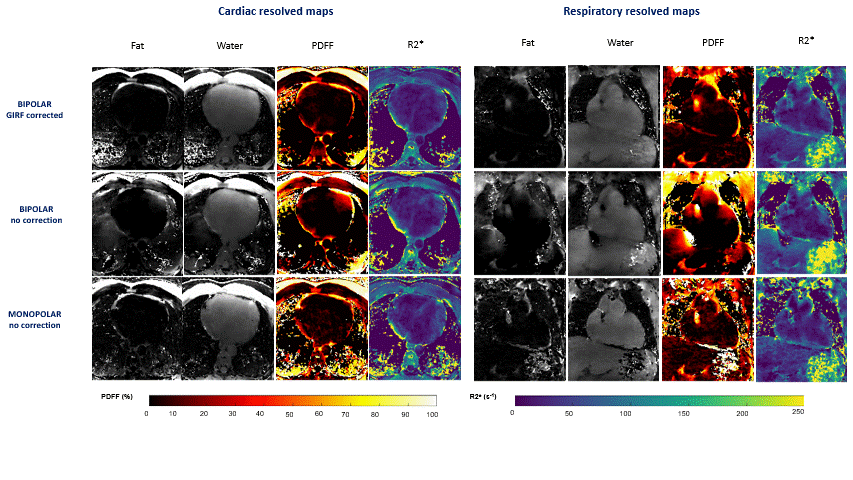

Supplement: Supplementary file 1 — Supplementary material. Fig. 1: Fat and water images with quantitative PDFF and R2*, B0 during the cardiac cycle in axial view resulting from bipolar with or without GIRF correction and monopolar echoes. [file mmc1.zip › Supplementary_Figure_1.gif]
